# Biological and morphological consequences of dsRNA-induced suppression of tetraspanin mRNA in developmental stages of *Echinococcus granulosus*

**DOI:** 10.1186/s13071-020-04052-y

**Published:** 2020-04-10

**Authors:** Seyed Mohammad Mousavi, Ali Afgar, Mohammad Ali Mohammadi, Seifollah Mortezaei, Ashkan Faridi, Balal Sadeghi, Majid Fasihi Harandi

**Affiliations:** 1 Research Center for Hydatid Disease in Iran, School of Medicine, Kerman University of Medical Sciences, Kerman, 7616914115 Iran; 2 Department of Parasitology, School of Medicine, Shahrekord University of Medical Sciences, Shahrekord, Iran; 3 Department of Food Hygiene and Public Health, Faculty of Veterinary Medicine, Shahid Bahonar University of Kerman, Kerman, Iran

**Keywords:** RNAi, Hydatid disease, Tegument, In vitro culture, Development

## Abstract

**Background:**

Cystic echinococcosis, caused by the cestode *Echinococcus granulosus*, is a neglected tropical disease with remarkable morbidity in humans and a problem of worldwide economic importance in livestock industry. Understanding the molecular basis of the parasite growth and development is essential for the disease diagnosis, management and control. The tetraspanin (TSP) family of proteins are transmembrane proteins with a role in many physiological processes of eukaryotic organisms. TSPs present in the tegumental surface of platyhelminths play pivotal roles in host-parasite interaction. However, little is known about the role of TSPs in growth and development in the Platyhelminthes. To understand the role of TSP1 in the growth and development of *E. granulosus* we investigated the effect of EgTSP1-specific long dsRNA in different in vitro stages of the parasite.

**Methods:**

Different stages of *E. granulosus*, protoscoleces and strobilated worms, were cultivated In vitro in di-phasic media. Using long dsRNA and two delivery methods, i.e. electroporation and electro-soaking, EgTSP1 silencing was performed with an EgTSP1-specific dsRNA. The TSP1 expression profile was assessed as well as the biological and ultrastructural properties of the parasites.

**Results:**

After three days of dsRNA treatment, EgTSP1 expression was significantly reduced in both stages of *E. granulosus* as compared to irrelevant/unrelated dsRNA and untreated controls. Silencing expression of EgTSP1 in different stages of *E. granulosus* resulted in reduced viability and body contractions, inhibition of protoscoleces evagination and distinctive tegumental changes. Ultrastructural morphology of the strobilated worms treated with EgTSP1-specific dsRNA was indicative of the microtriches impairments and vacuolated tegument compared to the control helminths.

**Conclusions:**

Results of the present study suggest that EgTSP1 plays important structural roles in tegument configuration in *E. granulosus*. EgTSP1 is proved to be a potential target for the development of vaccines and RNAi-based drugs.
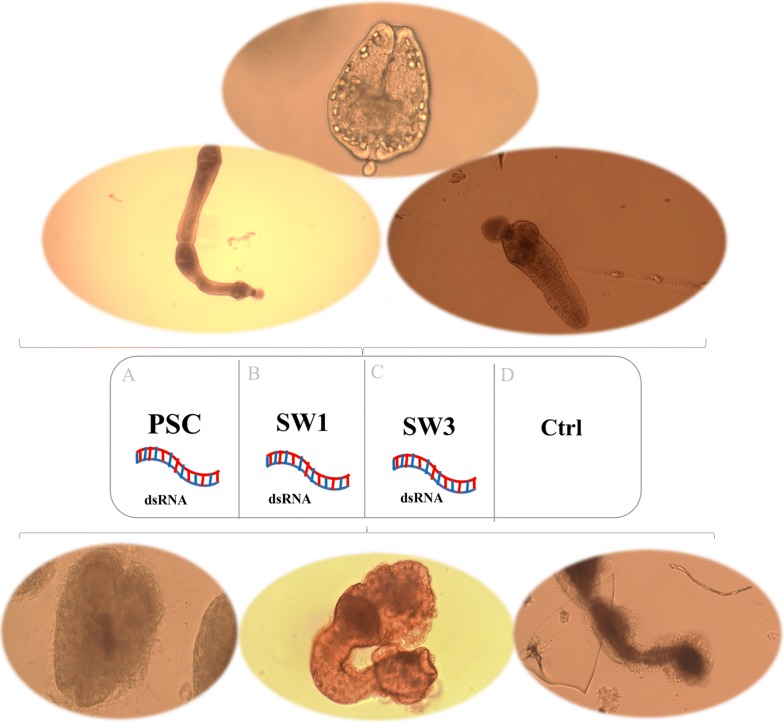

## Background

*Echinococcus granulosus* is a small canine tapeworm, whose larval stage causes cystic echinococcosis (CE) in humans and livestock. CE as a neglected zoonotic disease represents an economic and public health problem in many developing countries [1]. The natural life-cycle of *E. granulosus* typically includes dogs as the definitive hosts and domestic and wild ungulates as the intermediate hosts. The adult worms are localized in dog small intestine, where eggs are excreted with feces to the environment. Upon ingestion of the eggs, hatched oncospheres penetrate the intestinal mucosa and migrate through the blood and lymphatic system to virtually every organ predominantly in the liver and lungs. The parasite develops into a fluid-filled cyst, containing numerous metacestodes known as protoscoleces (PSCs) [2, 3].

The potential of the helminth to bidirectionally develop to either the strobilated worm in di-phasic culture media or to the microcysts in mono-phasic media, provides a valuable tool for understanding *Echinococcus* biology. Molecular interventions in the helminth cellular machinery improve our knowledge of the disease and can help develop future molecular tools for CE control and prevention. Furthermore, using *Echinococcus* as a model for invertebrate biology provides the foundation for future research on other platyhelminth species [3, 4].

RNA interference (RNAi) or post-transcriptional gene silencing (PTGS) has been successfully applied in numerous organisms for characterization of genes involved in regeneration and development and has provided valuable information on gene function [5, 6]. In recent years, RNAi has been used for a number of genes in parasitic helminths, leading to suppression of functional genes in the parasites. Several studies applied dsRNA-based interference technology on different helminth taxa including flatworms [7, 8], nematodes [9–11] and turbellarians [12]. Relatively few studies have been conducted on the use of dsRNAs in cestode parasites [13–15]. Silencing of the Me-act-1 gene in *Moniezia expansa* with dsRNA suppressed mRNA expression in the tegument, and resulted in increased tegumental disruption/blebbing and reduced F-actin levels in muscle adult worm [14].

Parasitic platyhelminths including *E. granulosus*, have a unique outer multinucleated syncytial surface (the tegument) containing tegumental cell bodies. Tegument is actively involved in different nutritional and osmoregulatory functions of platyhelminths [16, 17]. The functional importance of the tegument represents an obvious target for development of new anthelmintic products [18]. Through the application of various proteomics methods, the protein composition of the tegument is well understood [19, 20].

Among the proteins, are a group of membrane proteins called tetraspanins (TSPs). This family of proteins were first described by Parkinson et al. [22] and Hu et al. [21], who demonstrated the presence of TSPs in the outer tegument membrane of *E. granulosus.* TSPs are a large superfamily of plasma membrane-bound proteins that are thought to be present in all metazoans. Proteins of this family consist of four conserved transmembrane domains (TM1-TM4), cytoplasmic tails at the N- and C-terminal regions, and a small and large extracellular loop (EC-1 and EC-2, respectively) containing a Cys-Cys-Gly motif and 2–6 additional cysteines [23, 24].

TSPs are widely distributed in many cell types, but their precise physiological roles are not fully understood. TSPs participate in a broad spectrum of cellular activities, including cell proliferation, cell fusion, motility, adhesion, migration, sperm-egg fusion and signal transduction pathways [25, 26]. TSPs in trematodes have an important role in the development, maturation and stability of the tegument and are involved in the immune evasion of schistosomes [27, 28]. Recently, many studies have focused on some members of the TSPs family as a vaccine candidate against schistosomiasis [20, 28], alveolar echinococcosis [29, 30], filariasis [31] and clonorchiasis [32].

Despite their promise as vaccines, the functions of TSPs have not yet been elucidated. Hu et al. [21] have studied immunolocalization of *E. granulosus* TSP1 (EgTSP1) in different protoscoleces and adult stages of the parasite. Using EgTSP1-specific siRNA on protoscoleces resulted in thinner tegumental distal cytoplasm. However, Hu et al. [21] provided no data on the strobilated forms of the parasite. Looking into the molecular structure and function of the adult worms (in dogs) could potentially provide us necessary information and tools to combat the disease in human hosts. The potential of RNAi technology to target major structural proteins of tapeworms has been poorly investigated. While the TSPs family has been extensively studied and well characterized in several platyhelminths [20, 29, 30], there are very few data on genomic manipulation of TSPs by RNAi. Suppression of Sm-tsp1 and Sm-tsp2 mRNA in *S. mansoni* using dsRNA resulted in impairments in tegument integrity, maturation and stability [33]. The present study investigated the potential of dsRNA-mediated EgTSP1 suppression and its molecular and biological consequences in developmental stages of *E. granulosus in vitro*.

## Methods

### Parasite preparation and culture

*Echinococcus granulosus* cysts were obtained from livers of naturally infected sheep slaughtered under the supervision of the veterinary officers in Kerman municipal abattoir. The hydatid fluid containing PSCs was taken out with a 20 ml syringe and then the cyst membranes were removed. The protoscoleces were carefully washed three times in sterile phosphate-buffered saline (PBS) containing 100 U/ml penicillin and 100 μg/ml streptomycin. A minimum viability of 95% was considered as a threshold measured by 0.1% eosin exclusion test as previously described [34]. Protoscoleces were cultured in vitro as previously described with minor modifications [35]. Briefly, protoscoleces were separated from brood capsules by two layers of sterile gauze. After treating in 20% dog bile in CMRL 1066 for 24–48 h, the PSCs were used for In vitro culture in diphasic media. Strobilated worms were obtained in the cultures after 55 days [36].

### dsRNA synthesis, delivery and qPCR assays

Synthesis and purification of dsRNA were carried out using Megascript RNAi kit (Ambion, Austin, TX, USA) according to the manufacturer’s instructions. Full length (1065 bp) dsRNAs of EgTSP1 were synthesized from cDNA encoding *E. granulosus* tetraspanin1 (GeneDB: EgrG_002030400.1) by using gene-targeted primers containing T7 promoter sequences (EgTSP1F: 5′-TAA TAC GAC TCA CTA TAG GGA TGG GCA AGC GCA TTT CG-3′ and EgTSP1R 5′-TAA TAC GAC TCA CTA GAC TAG GGC ACT TTG TGC TTT TCC TTG A-3′). To confirm integrity, dsRNAs were electrophoresed by non-denaturing 1% agarose gel (Additional file 1: Figure S1). The concentration and purity were determined by a spectrophotometer (NanoDrop ND-1000; NanoDrop Technologies, Wilmington, DE, USA). To exclude secondary targets, we first aligned genome sequences of the entire TSP family and accurately examined the sites that were not conserved. Then, to evaluate secondary targets, we sequenced the whole genome in CLC software into small and random fragments. An unknown exogenous irrelevant dsRNA sequence from the expression vector pPIC9K was used as the negative control [37], kindly provided by Dr S. Faezi, Guilan University of Medical Sciences. The vector was used to generate the unrelated dsRNA.

The study was carried out on different developmental stages including protoscoleces (PSC), strobilated worm with one proglottid (SW1) and strobilated worms with three or more proglottids (SW3). The following treatments were included in the study: (i) EgTSP1-specific dsRNA delivered by electroporation; (ii) EgTSP1-specific dsRNA delivered by electro-soaking; (iii) irrelevant dsRNA as negative control delivered by electro-soaking; and (iv) no-treatment.

For dsRNA delivery using electroporation of the parasites within each group, 5000 protoscoleces, 20 strobilated worms with one proglottid and 20 strobilated worms with three or more proglottids, were separately submerged in 200 μl electroporation buffer (150 mM sucrose, 27 mM Na2HPO4, adjusted pH to 7.5) containing dsRNA in a final concentration of 50 nM and electroporated in a 2 mm cuvette by applying a time constant protocol with a single 20 ms impulse, at 125 V using Gene Pulser II (Bio-Rad, CA, USA). We used the minimum amount of RNAi (50 nm) to minimize secondary target effects [38]. After treatment, the parasites were maintained in 37 °C in 5% CO_2_ for 30 min and then were transferred to 6-well plates with fresh CMRL1066 medium without dsRNA.

For dsRNA delivery using electro-soaking, electroporation was carried out as described above and each group was separately cultured in 2.5 ml CMRLL1066 culture media containing 50 nM dsRNAs and a laboratory-made transfection reagent [39] was then added to each well and were maintained for 24 h. Long dsRNA delivery was repeated on 3, 7 and 14 days after the initial transfection.

At the end of the experiment, the total RNA was extracted using RNeasy Mini Kit (Qiagen, Hilden, Germany) according to the manufacturer’s instructions. The concentrations of the extracted RNA were adjusted using ND-1000 spectrophotometer (Nano Drop Technologies). Reverse transcription was performed using PrimeScript RT reagent kit (Takara, Otsu, Shiga, Japan).

Quantitative Real-time PCR was performed using a Rotor-Gene 6000 Q (Qiagen) according to SYBR Premix Ex Taq^TM^ II instructions (Takara). The sense and antisense primers specifically designed for EgTSP1 were TSP1F 5′-CGG GAA TGA GAG TGT GGA GGG-3′ and TSP1R 5′-CCT CGT AGC CAT CCA TGC CG-3′. Beta-actin was amplified as an internal housekeeping reference gene using RF 5′-ATG GTT GGT ATG GGA CAA AAG G-3′ and RR 5′-TTC GTC ACA ATA CCG TGC TC-3′ as forward and reverse primers, respectively. All real-time PCR experiments were performed in duplicate in a total reaction volume of 10 µl containing 2 µl cDNA target, 5 µl SYBR Premix Ex Taq^TM^ II, 0.4 μM primers and 2 μl RNase-free water. Standard cycling conditions were as follows: 95 °C for 30 min, 40 cycles of 94 °C for 10 s, 60 °C for 18 s, and 72 °C for 20 s. The relative gene expression levels were calculated using the 2^−∆∆Cq^ method. TSP-1 expression level in the invaginated protoscoleces was used as the basic level for comparing different stages.

### Survival, strobilization and phenotype study

Following dsRNA treatment, the parasites were microscopically examined every day for 21 days, and viability and evagination of the PSCs, morphological changes, strobilization and motility of the strobilated worms were evaluated. PSCs viability was assessed by flame cell activity and 0.1% eosin exclusion test. Also, the number of evaginated PSCs per 100 PSCs was assessed under a light microscope. The motility of the strobilated worms was evaluated by measuring the number of body contractions per minute. Strobilization in one-proglottid worms was monitored until day 14 post-treatment. On days 3, 7, 14 and 21, worms with three or more proglottids were fixed in 3% glutaraldehyde in 0.1 M phosphate buffer, pH 7.4, for transmission electron microscopy (TEM).

### Statistical analysis

All data are presented as the mean ± standard error (SE). Statistical comparisons between and within groups were performed by ANOVA using the software package GraphPad Prism 6 (www.graphpad.com). *P*-values of less than 0.05 were considered significant.

## Results

### Parasite cultivation

*Echinococcus granulosus* PSCs were successfully developed to strobilated worms in di-phasic medium. Developmental stages included intact protoscoleces (day 0), evaginated protoscoleces (day 1), excretory canal formation (day 7), first proglottid formation (day 28), second proglottid formation (day 40), and 3 or more proglottids formation (observed after 55 days of cultivation). The parasite was characterized as *E. granulosus* (*sensu stricto*) G1 genotype using *cox*1 PCR-sequencing (GenBank: MG832791).

### EgTSP1 expression profile after dsRNA treatment

**Fig. 1:**
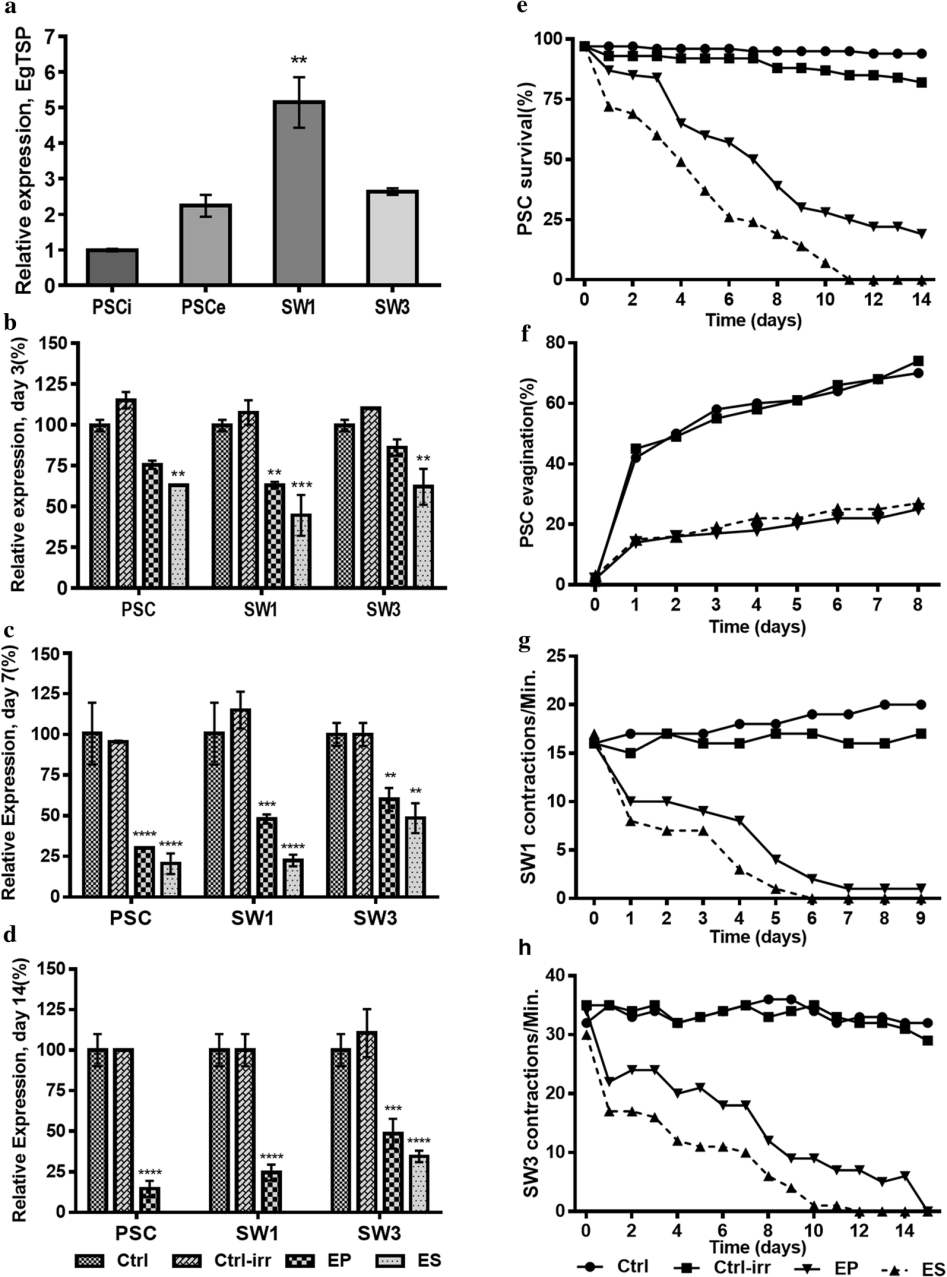
Molecular (**a**–**d**) and biological (**e**–**h**) changes induced by tetraspanin1(EgTSP1)-specific dsRNA in different developmental stages of *Echinococcus granulosus* using electro-soaking (ES) and electroporation (EP) compared to the untreated (Ctrl) and the irrelevant dsRNA (Ctrl-irr) controls. **a** EgTSP1 expression profile in different developmental stages of *E. granulosus in vitro*; invaginated protoscoleces (PSCi), evaginated protoscoleces (PSCe), one-proglottid worms (SW1) and three or more proglottids worms (SW3). **b**–**d** Suppression of EgTSP1 in protoscoleces (PSC), one-proglottid worms (SW1) and three or more proglottids worms (SW3) by dsRNA on days 3, 7 and 14. **e** Viability changes of protoscoleces treated with EgTSP1-specific dsRNA. **f** Evagination rate of protoscoleces treated with EgTSP1-specific dsRNA. **g**, **h** Changes in body contractions per minute in one-proglottid worms (SW1) and three or more proglottids worms (SW3) treated with EgTSP1-specific dsRNA. For Ctrl-irr EgTSP1 expression data are only demonstrated for electro-soaking method. Bars show the mean ± SE. *P < 0.05, **P < 0.01, ***P < 0.001, ****P < 0.0001

Figure 1a shows EgTSP1 expression in different developmental stages of *E. granulosus*. Significantly higher levels of EgTSP1 expression were detected in one-proglottid worm group (SW1) compared to the other groups. The relative EgTSP1 expression in developmental stages of *E. granulosus* treated by EgTSP1-specific dsRNA on days 3, 7, 14, respectively, is shown in Fig. 1b–d. In all developmental stages, significantly reduced levels of EgTSP1 expression were recorded on day 3 after dsRNA treatment with electro-soaking compared to the irrelevant negative dsRNA and untreated control (Fig. 1b, Table 1) (ANOVA: *F*_(3, 12)_*=* 49.97, *P *< 0.0001). The efficacy of dsRNA silencing was method-dependent, and the most effective silencing was observed in electro-soaking with 51–80% of EgTSP1 mRNA suppression in the treated parasites after 7 days. Using electroporation, after 7 days 40–64% suppression of EgTSP1 mRNA were observed as compared with irrelevant negative siRNA and untreated control (Fig. 1c) (ANOVA: *F*_(3, 12)_*= *81.50, *P *< 0.0001). Following 21-day RNA interference, 68 and 53% EgTSP1 expression suppression were observed in 3 or more-proglottids worm group (SW3) using electro-soaking and electroporation respectively (Table 1).

**Table 1:** Expression suppression (%) induced by tetraspanin 1 (EgTSP1)-specific dsRNA in different developmental stages of *Echinococcus granulosus* using electro-soaking and electroporation

Transfection	Electro-soaking				Electroporation			
Day	3	7	14	21	3	7	14	21
PSC	38	80	nd	nd	25	64	85	nd
SW1	55	77	nd	nd	37	52	75	nd
SW3	38	51	65	68	14	40	51	53

*Abbreviations*: nd, not determined (the parasites were degenerated and/or dead)

### Morphological and physiological changes

**Fig. 2:**
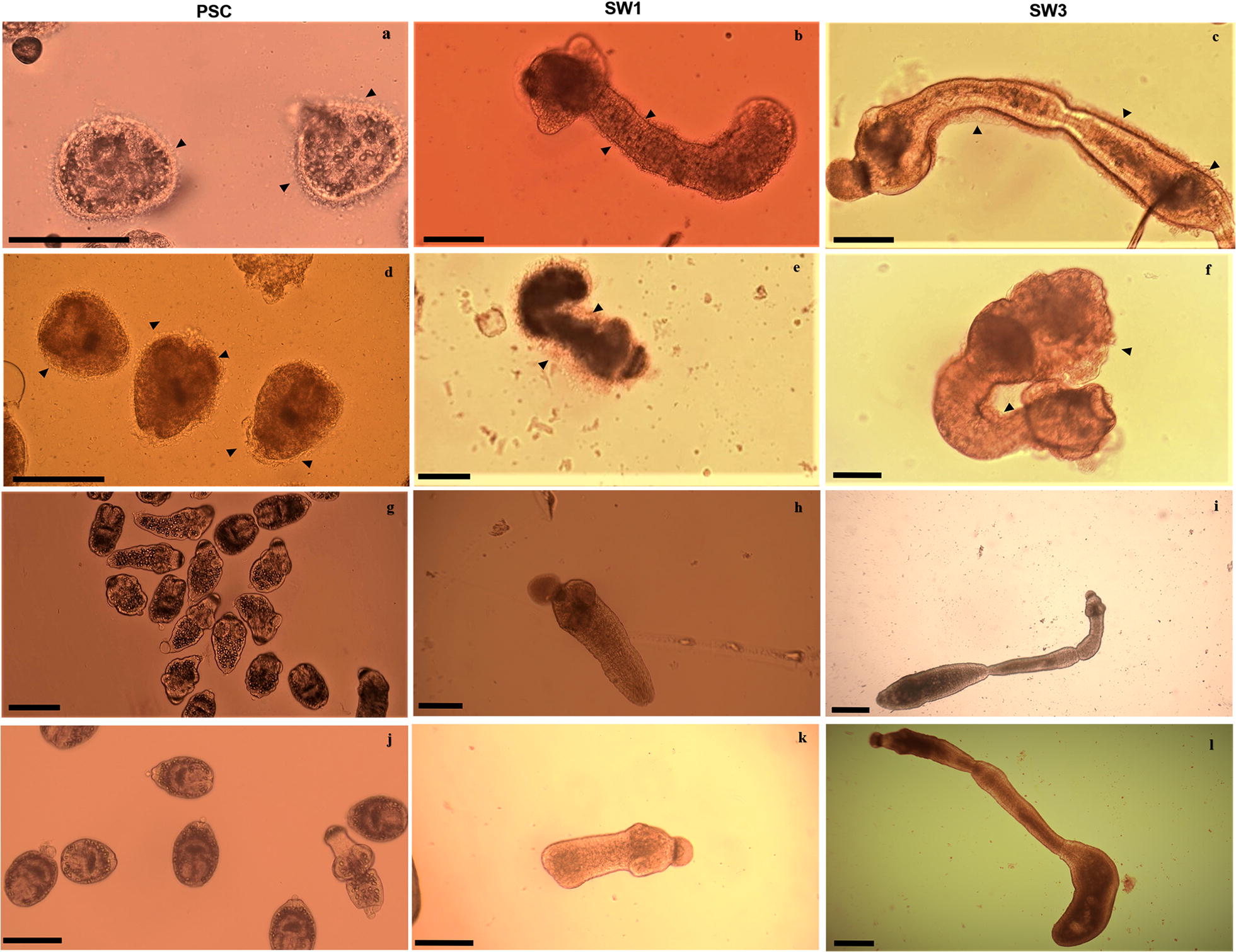
Morphological changes of *Echinococcus granulosus* following In vitro treatment with tetraspanin1 (EgTSP1)-specific dsRNA. Different development stages, protoscoleces (PSC, left column), one-proglottid worms (SW1, middle column) and three or more proglottids worms (SW3, right column) were treated by the dsRNA using electroporation (**a**, **b**, **c**), and electro-soaking (**d**, **e**, **f**) as compared to the irrelevant dsRNA (**g**, **h**, **i**) and untreated controls (**j**, **k**, **l**). Note the outer layer irregularities and malformations (arrowheads) in the tegument of protoscoleces (PSCs) and strobilated worms (SW). *Scale-bars*: 200 μm

Protoscoleces viability and evagination rates are shown in the Fig. 1e, f. The viability of dsRNA-treated PSCs after 7 days was decreased to 24%, and after 11 days no viable PSCs were observed compared to the control groups (ANOVA: *F*_(3, 56)_ = 32.40, *P* < 0.0001). The evagination rate of the PSCs after 8 days of dsRNA treatment was 25% while the irrelevant siRNA and untreated control groups demonstrated a 74% and 70% evagination rate, respectively (Fig. 1f) (ANOVA: *F*_(3, 32)_ = 14.34, *P* < 0.0001). As shown in Fig. 2, tegumental alterations were detected in dsRNA-treated PSCs in comparison with the control groups.

For the strobilated stages (SW1 and SW3) the morphology as well as the body contractions and strobilization were found to be altered between the treatment and control groups. In one-proglottid worm group (SW1), no body contraction was observed six days after EgTSP1 silencing (Fig. 1g) (ANOVA: *F*_(3, 36)_ = 32.69, *P* < 0.0001), while 18 body contractions per minute was recorded in irrelevant dsRNA and untreated controls.

Our results suggested that EgTSP1 may play a role in strobilization in the course of helminth development. While after 2 weeks, strobilization was observed in all helminths of the irrelevant dsRNA and untreated control groups, no further strobilization was observed in one-proglottid worms treated by EgTSP1 dsRNA. For three or more proglottids group, body contraction was significantly reduced in both transfected groups. On day 8 post-treatment helminths of the three or more proglottids group demonstrated 33 and 35 contractions/min in irrelevant dsRNA and untreated controls respectively (ANOVA: *F*_(3, 60)_ = 64.13, *P* < 0.0001). The corresponding numbers of contractions were 6 and 12 for electro-soaking and electroporation groups respectively (Fig. 1h).

**Fig. 3:**
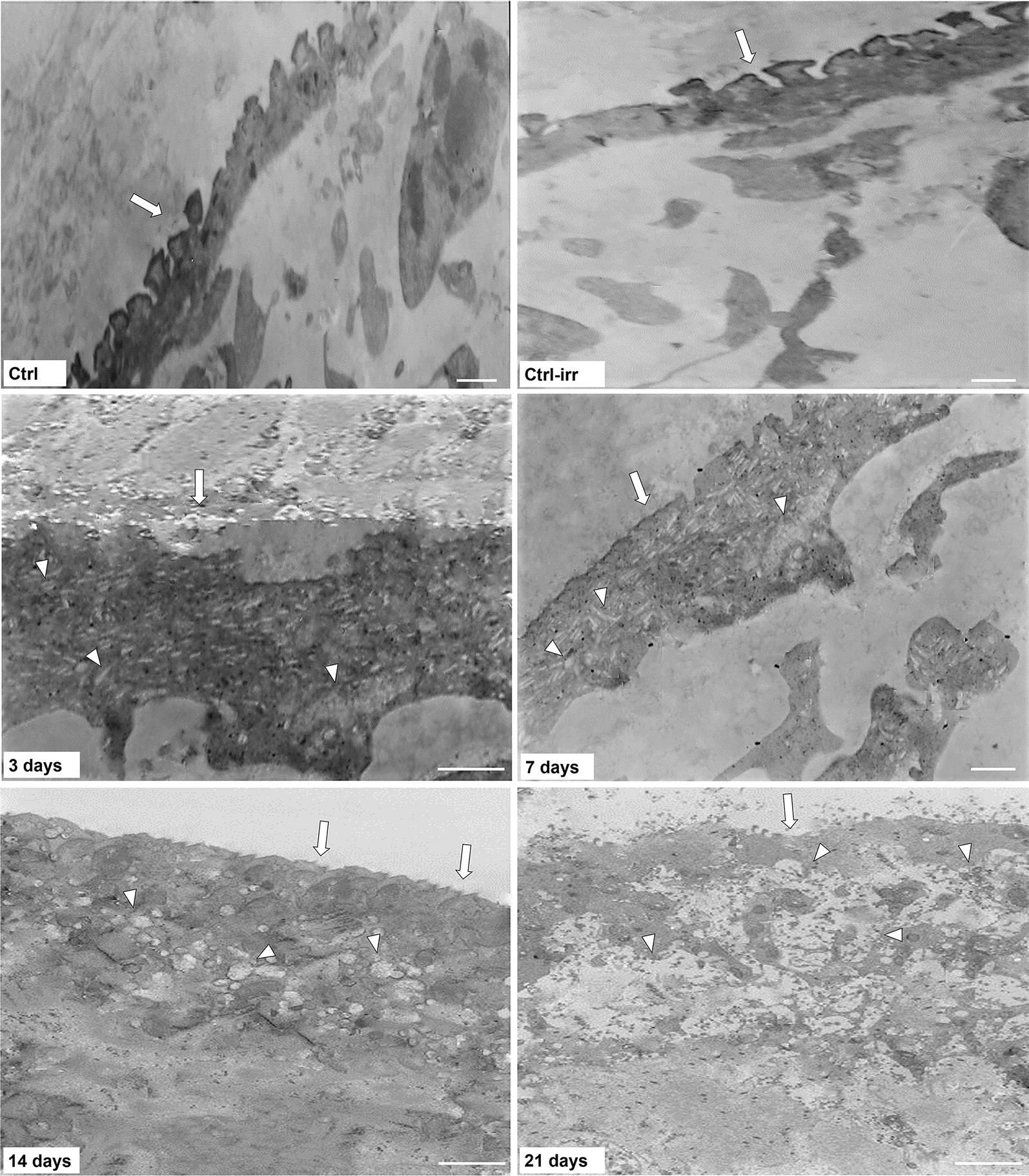
Transmission electron microscopy (TEM) micrograph of the of *Echinococcus granulosus* tegument following EgTSP1-specific dsRNA. Tegumental changes in three or more proglottids worms treated by EgTSP1-specific dsRNA on days 3, 7, 14 and 21 as compared to irrelevant dsRNA (Ctrl-irr) and untreated Control (Ctrl). Note the impaired microtriches and surface damages (arrows) and marked vacuolations (arrowheads). *Scale-bars*: 2 μm

As shown in Fig. 2, EgTSP1 suppression resulted in remarkable morphological changes and irregularities in the surface structure of the strobilated worms as compared to the controls with a smooth outer surface. Tegumental structure of the three or more proglottids worm group treated with EgTSP1 dsRNA was studied using TEM. EgTSP1 silencing in strobilated worms resulted in impaired microtriches and small vacuoles developed in the tegumental cytoplasm after three days. Twenty-one days after dsRNA treatment some ultrastructural changes were noted including the presence of enlarged extensive vacuoles, empty internal cavities and remarkable surface damage in the tegumental cytoplasm (Fig. 3).

## Discussion

In the present study EgTSP1 suppression in *E. granulosus* was attempted using RNAi technology. Findings of this study indicated molecular and biological consequences in different developmental stages following dsRNA interference. Platyhelminths express a family of TSP in their tegument and some of the TSP members are candidates of vaccine development against platyhelminth infections [19, 20, 30]. There are currently at least 33 members of the TSP superfamily known in humans or mammals, 20 in *Caenorhabditis elegans*, 36 in *Drosophila melanogaster*, 29 in *Schistosoma* spp. and 30 in *E. granulosus* [40, 41]. Although the TSP family has been extensively studied and well characterized in parasitic organisms there are very few studies on the TSP functions and its biological properties in helminth parasites. A few studies have used RNAi to suppress a few *Echinococcus* genes to determine their function. Using small interfering RNA (siRNA), thioredoxin peroxidase (TPx) gene silencing was achieved in the PSCs and led to the impairment of development in the parasite [15]. In another study, calmodulin expression was suppressed by siRNA treatment in in vitro-cultured *E. granulosus* and consequently the parasites exhibited morphological and biological changes as well as lowered viability rate [36]. Hu et al. [21] have used EgTSP1-specific siRNA for silencing the gene and specifically reduced the level of EgTSP1 expression by 64% in the PSCs accompanied by pronounced phenotypic changes in the tegument. To understand the role of TSP1, we explored the effects of EgTSP1-specific long dsRNA in different developmental stages of *E. granulosus*.

According to the findings of the present study EgTSP1 mRNAs were expressed throughout the development from the PSCs to the strobilated worms with the highest expression in one-proglottid worms (Fig. 1a). To our knowledge, no comparative study has been carried out to evaluate TSP expression between different developmental stages of a single clone of *E. granulosus*. However, several studies have investigated TSP expression in trematodes, a closely related platyhelminth group. Tran et al. [33] showed Sm-tsp1 and Sm-tsp2 were highly expressed in cercariae and eggs of *S. mansoni*, respectively. In *Opisthorchis viverrini* the highest expression of Ov-tsp-2 and Ov-tsp-3 was observed in the eggs [42]. In contrast, Piratae et al. [27] found highly expressed Ov-tsp-1 in the metacercariae of this species.

Our experiments showed that in PSCs on day 3, EgTSP1 expression was reduced significantly by 38% (Fig. 1b, Table 1). Hu et al. [21] successfully suppressed TSP1 genes expression by 61% in PSCs. In *S. mansoni*, Tran et al. [33] found the highest TSP suppression in the adult worms and schistosomula of *S. mansoni* at 7 days post-treatment (61% and 75% suppression, respectively). We demonstrated that silencing of EgTSP1 expression affects the viability and evagination of the PSCs (Fig. 1e, f). TSP1 suppression exerted a significant effect on the viability of protoscoleces. In addition, the process of evagination was significantly slowed down after dsRNA treatment, i.e. 74% of irrelevant dsRNA control PSCs were evaginated while evagination was observed in only a quarter of the protoscoleces in the treatment groups. There are no studies relevant to the biological consequences of RNAi-induced TSP1 suppression in platyhelminths; however, several studies have been conducted on other genes including thioredoxin peroxidase, calmodulin, 14-3-3 and elp [13]. For thioredoxin peroxidase (TPx), siRNA treatment in the PSCs led to the reduced viability by 79% compared to the untreated control [15]. In another study, it has been shown that the viability of the PSCs was significantly affected by EgCaM-specific siRNA three days post-treatment [36]. The present findings indicate a crucial role for TSP1 gene in developmental biology and differentiation of *E. granulosus* protoscoleces.

Targeting TSP1 by using specific dsRNA induced similar effects on the In vitro cultured strobilated worms. Significant reductions in EgTSP1 expression were observed three days after dsRNA treatment (Table 1). Following EgTSP1 suppression we found reduced body contractions in the strobilated worms compared to untreated control groups (Fig. 1g, h). Interestingly no further strobilization was observed in one-proglottid worms treated by TSP1-specific dsRNA. No other data are available on the effects of TSP1 suppression in the strobilated forms of *E. granulosus*. In two studies on adult worms of *O. viverrini*, treatment by specific dsRNAs, resulted in expression suppression of Ov-tsp-1, Ov-tsp2 and Ov-tsp-3 [27, 42].

The suppression induced by RNAi has been demonstrated also in several other genes in parasitic worms [9, 36, 43–45]. Guidi et al. [44] showed Ca2+-dependent protein kinases play a key role in the viability and motility of the adult worms and schistosomula of *S. mansoni*. Mousavi et al. [36] showed calmodulin-specific siRNA decreases motility and strobilar contractions in *E. granulosus* after two days of treatment [36].

Silencing EgTSP1 led to tegumental changes in all developmental stages *E. granulosus* (Fig. 2). Similar findings have been reported in previous studies on TSP family in other helminth species including adult worms of *S. mansoni* [33] and *O. viverrini* [27, 42], as well as cuticle dissociation in the free-living nematode *C. elegans* [46]. The present findings indicate that the TSP family might play an important role in the biogenesis of the tegument.

TEM findings for three or more proglottids worm group indicated phenotype changes including impaired microtriches, vacuolization and surface damages in the tegumental cytoplasm after three days of dsRNA treatment (Fig. 3). In *O. viverrini*, silencing of three TSP members resulted in thinner and largely vacuolated tegument [27, 42]. The tegumental distal cytoplasm of tsp-2-specific dsRNA-treated schistosomula and adult worms of *S. mansoni* was highly vacuolated and much thinner than that of the luciferase dsRNA control [33]. Our TEM results are in line with those of Hu et al. [21] in which silencing EgTSP1 in the PSCs led to thinner tegument with malformations and vacuolization.

The tegument is a syncytial layer that covers the whole worm with a plasma membrane as an interface of host-parasite interaction. The tegument of platyhelminths plays an essential role in different processes of parasite development. This coating is actively involved in the absorption of food, digestion, synthesis and secretion of substances, osmoregulation, waste disposal, and the protection of the helminth against enzymes and host’s immune system. Microtriches, the processes extending to the outermost surface of cestode tegument, are essential elements for increasing the absorption surface [16, 17].

Several tegument surface proteins exposing to the host have been identified in a number of proteomic studies in platyhelminths [47–49]. Some membrane-bound proteins have been demonstrated, including important proteins such as binding proteins, heat-shock proteins, enzymatic and transmembrane proteins [50]. Sotillo et al. [51] showed the expression profiles of tegument proteins upregulated over 3 h to 5 days during transformation of cercariae to schistosomula in *S. mansoni*. Surface proteins of the tegument are ideal targets for vaccine development and drug design. Dang et al. [30] characterized seven tetraspanins (TSP1-TSP7) in *E. multilocularis* metacestodes and showed a significant reduction of cystic lesions in BABL/c mice infected with *E. multilocularis* indicating immunogenic properties of TSP1 and TSP3 as vaccine candidates.

The present findings have potential implications for echinococcosis control in humans and animals. However further in-depth studies are required to elucidate the mechanisms underlying biological and morphological changes following TSP silencing and to determine which fragment(s) or portion(s) of EgTSP1-specific dsRNA are useful as potential therapeutic agents against echinococcosis.

## Conclusions

We used long dsRNA for suppressing EgTSP1 in protoscoleces and strobilated worms of *E. granulosus*. We demonstrated the significant effect of dsRNA treatments on the gene expression as well as the phenotype *E. granulosus*. The study suggests EgTSP1 as an important gene for viability and development of *E. granulosus* and as a fundamental element in tegument configuration and biogenesis.

## Supplementary information

**Additional file 1: Figure S1.** Agarose gel electrophoresis of EgTSP1-specific dsRNA produced in *Echinococcus granulosus.* Lane A: ladder; Lane B: control (500 bp); Lane C: dsRNA (1065 bp).

## Data Availability

Data supporting the conclusions of this article are included within the article and its additional file. The raw data will be made available upon request.

## Abbreviations

PSCs: Protoscoleces
SW: Strobilated worms
EgTSP1: *Echinococcus granulosus* tetraspanin 1
ES: Electro-soaking
EP: Electroporation
SE: Standard error
