# Supplementary material for: Biological and morphological consequences of dsRNA-induced suppression of tetraspanin mRNA in developmental stages of Echinococcus granulosus
Source: Parasit Vectors. 2020 Apr 10;13:190. doi: 10.1186/s13071-020-04052-y (PMC7146954; doi:10.1186/s13071-020-04052-y)
Supplement: Supplementary file 1 — Additional file 1: Figure S1. Agarose gel electrophoresis of EgTSP1-specific dsRNA produced in Echinococcus granulosus. Lane A: ladder; Lane B: control (500 bp); Lane C: dsRNA (1065 bp). [file 13071_2020_4052_MOESM1_ESM.docx]

**B**

**C**

**A**

**A**

**100**

**1065**

**500**


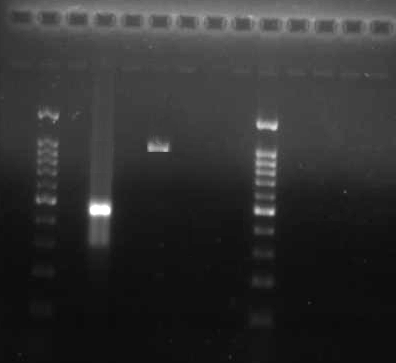


**b**

**c**

**a**

**a**

**100**

**1000**

**500**

**Additional file 1:** Figure S1. Agarose gel electrophoresis of EgTSP1-specific dsRNA produced in *Echinococcus granulosus.* A: Ladder. B: Control (500bp) C: dsRNA(1065bp)
